# Angioimmunoblastic T-cell lymphoma and correlated neoplasms with T-cell follicular helper phenotype: from molecular mechanisms to therapeutic advances

**DOI:** 10.3389/fonc.2023.1177590

**Published:** 2023-04-26

**Authors:** Luís Alberto de Pádua Covas Lage, Hebert Fabricio Culler, Cadiele Oliana Reichert, Sheila Aparecida Coelho da Siqueira, Juliana Pereira

**Affiliations:** ^1^ Department of Hematology, Hemotherapy & Cell Therapy, University of São Paulo (USP), São Paulo, SP, Brazil; ^2^ Laboratory of Medical Investigation in Pathogenesis and Directed Therapy in Onco-Immuno-Hematology (LIM-31), University of São Paulo (USP), São Paulo, SP, Brazil; ^3^ Department of Pathology, University of São Paulo (USP), São Paulo, SP, Brazil; ^4^ Department of Hematology and Oncology, Hospital Alemão Oswaldo Cruz (HAOC), São Paulo, SP, Brazil

**Keywords:** angioimmunoblastic T-cell lymphoma (AITL), T-cell follicular helper phenotype (TFH), epigenetic dysregulation, *RhoA* G17V mutation, *immunodysplastic syndrome*, hypomethylating agents (HMAs), histone deacetylase inhibitors (HDAi)

## Abstract

Angioimmunoblastic T-cell lymphoma (AITL) is the second most frequent subtype of mature T-cell lymphoma (MTCL) in the Western world. It derives from the monoclonal proliferation of T-follicular helper (TFH) cells and is characterized by an exacerbated inflammatory response and immune dysregulation, with predisposition to autoimmunity phenomena and recurrent infections. Its genesis is based on a multistep integrative model, where age-related and initiator mutations involve epigenetic regulatory genes, such as *TET-2* and *DNMT3A*. Subsequently, driver-mutations, such as *RhoA* G17V and *IDH-2* R172K/S promote the expansion of clonal TFH-cells (“second-hit”), that finally begin to secrete cytokines and chemokines, such as IL-6, IL-21, CXCL-13 and VEGF, modulating a network of complex relationships between TFH-cells and a defective tumor microenvironment (TME), characterized by expansion of follicular dendritic cells (FDC), vessels and EBV-positive immunoblasts. This unique pathogenesis leads to peculiar clinical manifestations, generating the so-called “*immunodysplastic syndrome*”, typical of AITL. Its differential diagnosis is broad, involving viral infections, collagenosis and adverse drug reactions, which led many authors to use the term “*many-faced lymphoma*” when referring to AITL. Although great advances in its biological knowledge have been obtained in the last two decades, its treatment is still an unmet medical need, with highly reserved clinical outcomes. Outside the setting of clinical trials, AITL patients are still treated with multidrug therapy based on anthracyclines (CHOP-like), followed by up-front consolidation with autologous stem cell transplantation (ASCT). In this setting, the estimated 5-year overall survival (OS) is around 30-40%. New drugs, such as hypomethylating agents (HMAs) and histone deacetylase inhibitors (HDAi), have been used for relapsed/refractory (R/R) disease with promising results. Such agents have their use based on a biological rationale, have significant potential to improve the outcomes of patients with AITL and may represent a paradigm shift in the therapeutic approach to this lymphoma in the near future.

## 1 Introduction

Angioimmunoblastic T-cell lymphoma (AITL) is a rare mature T-cell lymphoid malignancy characterized by an intense inflammatory reaction and immune dysregulation, with a broad spectrum of clinical manifestations and distinct molecular findings (1–4). Its normal counterpart is the T-follicular helper cell (TFH), a subtype of effector T-lymphocyte that plays a crucial role in the activation of B-cells and in their differentiation within the germinal center (2–4). Under physiological conditions, TFH-lymphocytes actively regulate the maturation of centroblasts into centrocytes, and subsequently, their differentiation into memory B-cells and plasma cells, in addition to ensuring homeostasis of immune tolerance mechanisms. Therefore, the clonal proliferation of TFH-cells generates an imbalance in the germinal center, characterized by pro-inflammatory phenomena, autoimmunity and hypersecretion of immunoglobulins, biological hallmarks of AITL (4–6).

Classically, mature T-cell lymphomas (MTCL), previously known as peripheral T-cell lymphomas (PTCL), derive from activated, post-thymic T-lymphocytes and are classified according to their dominant clinical presentation into predominantly nodal (nMTCL), extranodal (enMTCL), primary cutaneous (pcMTCL), and disseminated or leukemic (lMTCL) (7–9). AITL is the second most frequent subtype of nMTCL, accounting for 10-15% of all MTCL (2, 10). The 5th. edition of the Classification of Hematopoietic and Lymphoid Tissue Neoplasms proposed by the World Health Organization in 2022 (WHO-HAEM5) recognizes AITL as a clinical-pathological entity that belongs to the group of nodal lymphomas with T-cell follicular helper phenotype (nTFHL) (11). This family comprises three main entities that share the gene signature and antigenic expression of TFH-cells, including the angioimmunoblastic-type nTFHL (nTFHL-AI); the follicular-type nTFHL (nTFHL-F), associated with recurrent chromosomal rearrangement t(5;9)(q33;q22) - *ITK/SYK*; and nTFHL not otherwise specified (nTFHL-NOS). Although nTFHL-AI is the prototype of these tumors, great overlap in clinical, immunophenotypic and molecular-genetic features exists among these entities (11–13).

Described by Frizzera et al. in 1974 as a new lymphoma-like disease affecting the elderly, with a clinical-pathological pattern similar to the graft-versus-host reaction, with immunological abnormalities and a frequently fatal course, AITL was initially called “*angioimmunoblastic lymphadenopathy with dysproteinemia*” (14). Subsequently, other authors reported series of similar cases under the designations “*immunoblastic lymphadenopathy*” and “*lymphogranulomatosis X*” (15, 16). Although most cases had a recurrent and fatal course, it was initially considered as a non-neoplastic hyperimmune reaction triggered by an unknown stimulus. However, the clonal nature of AITL was definitively demonstrated in the 1980s by studies that revealed recurrent chromosomal abnormalities and monoclonal pattern rearrangement of the T-cell receptor (TCR) gene in tissue samples from its patients (17, 18). This led to its recognition as a subtype of PTCL by the *Revised European American Lymphoma Classification* (REAL) in 1994 (19).

In the 2000s, studies involving gene expression profiling (GEP) determined that the normal counterpart of AITL was the TFH-cell (4, 6, 20). After 2010, different groups have described recurrent and inactivating mutations of epigenetic regulatory genes, involved in DNA-methylation and histone-deacetylation, in tumor samples from AITL patients. These findings have contributed significantly to understanding the biology of AITL and suggest a central role for recurrent somatic mutations involving epigenetic modulation in nTFHL oncogenesis (21–23). Finally, in 2014, the G17V somatic mutation of the *RhoA* gene (*Ras-homolog family member A*) was described as a molecular biomarker of AITL, being found in up to 60-70% of MTCL cases with TFH-phenotype (24, 25).

## 2 Epidemiology

AITL is a rare lymphoproliferative disorder, accounting for 1-2% of all non-Hodgkin’s lymphomas (NHL) and 15-20% of MTCL (3). According to the *International Peripheral T-cell and natural-killer/T-cell Lymphoma Project* (ITCLP), a pioneering study that described the pathological characteristics and clinical outcomes of more than 1,300 patients with MTCL from different regions of the world, AITL was the second most prevalent subtype of MTCL in the Western world, just behind peripheral T-cell lymphoma, not otherwise specified (PTCL, NOS) (10). Characteristically, AITL presents an inverse geographical tropism to other subtypes of T-cell lymphomas, being more common in Europe, particularly in the British Islands and Nordic countries, comprising 28.7% of MTCL, and less prevalent in Asia and North America, where it accounts for 17.9% and 16.0% of T-cell lymphomas, respectively (10). In the United States, its incidence is low, with 0.05 new cases per 100,000 inhabitants per year (26). On the other hand, epidemiological data from the *French National Cancer Agency* pointed to AITL as the most prevalent subtype of MTCL, accounting for 36.1% of cases diagnosed between 2010-2013 (27).

Epidemiological data from a subgroup analysis of the prospective *T-Cell Project* study, involving 282 AITL patients recorded between 2006 and 2018, revealed lower prevalence of this lymphoma in South America compared to other geographic regions (7% of MTCL, p=0.002) (28). This prevalence was similar to that reported by our group, which demonstrated 10.4% of AITL cases among 124-Brazilian patients with nMTCL diagnosed and treated at the Hematology Service of the University of São Paulo between 2000 and 2019 (29). This observation supports that regional differences in the incidence of AITL may indeed exist, which can be explained in part by the high incidence of other MTCL subtypes in Central and South America, such as extranodal NK/T-cell lymphoma (ENKTL) and adult T-cell leukemia/lymphoma (ATLL), both subtypes associated with oncovirus endemic in these areas of the globe.

AITL preferentially affects elderly patients, with a median age of 60 to 65 years. Although there is no clear predilection for gender and ethnicity, some studies indicate a slight predominance in Caucasian males (10, 28–30).

## 3 Etiopathogenesis

The oncogenesis of AITL occurs in an integrative model characterized by close interaction among neoplastic cells, the tissue immune microenvironment (TIME) and Epstein-Barr virus (EBV) (4, 31, 32). AITL originates from the neoplastic transformation of TFH cell, a subtype of T-CD4+ effector lymphocytes that reside in the germinal center and are characterized by high expression of the chemokine receptor CXCR5 (*C-X-C motif receptor 5*), chemokine CXCL-13 (*C-X-C motif ligand 13*), ICOS (*CD28-related inducible T-cell co-stimulator*), CD154, CD40L and NFATC1 (6, 33–36). TFH-cells participate in the formation of the germinal center, leading to the expansion of lymphoid B-cells and promoting their differentiation into plasma cells and memory B-cells, as well as promoting the proliferation of follicular dendritic cells (FDC). These phenomena are regulated by the secretion of CXCL-13 and IL-21 by the TFH-cells (31, 32, 37).

Under normal conditions, naïve T-CD4+ lymphocytes interact with dendritic cells in the germinal center, promoting the activation of ICOS protein on T-cells and consequently activation of the PI3K pathway, leading to up-regulation of BCL-6, a critical transcriptional factor for TFH-cell differentiation (38, 39). Subsequently, activated T-cells will overexpress PD-1 and CXCR5, transforming into precursor TFH-cells, which will migrate to the periphery of the follicle to interact with antigen-specific B-cells. This interaction promotes a reaction in the germinal center that leads to the terminal maturation of TFH-cells, with activation of the JAK-STAT pathway through the secretion of IL-6 and IL-21 (32, 40).

The TIME elements represent the majority of the cellular component in a lymph node involved by AITL. Similar to classic Hodgkin’s lymphoma (cHL), reactive T-lymphocytes, B-lymphocytes, plasma cells, macrophages, dendritic cells, and endothelial cells make up the AITL’s tumor microenvironment. Such cells promote an exacerbated inflammatory response and immune dysregulation, considered as biological hallmarks of AITL and responsible for the recurrent infectious and autoimmune complications observed in this tumor. The exacerbated secretion of CXCL-13 and VEGF-1 (*vascular endothelial growth factor-1*) by mutated TFH-cells promote the expansion and proliferation of FDC and high endothelial venules (HEV) commonly found in AITL (31). Studies in animal models demonstrated that in AITL, B-cells are retained for a longer time in the germinal center and have a greater opportunity to interact with TFH-cells. IL-21 secretion by TFH-cells induce expansion of B-lymphocytes, which terminally differentiate into immunoglobulin-secreting plasma cells. Polyclonal plasmacytosis recurrently occurs in tissues affected by AITL, and polyclonal gammopathy is a characteristic laboratory finding of this neoplasm (31, 41). Dendritic cells and macrophages of the tumor microenvironment are hyperactivated and secrete high levels of IL-6, an agent with pro-inflammatory and pro-proliferative activity on the TFH malignant clone (4).

The Epstein-Barr virus (EBV) is an oncogenic infectious agent implicated in the development of several subtypes of lymphomas. In up to 80% of AITL cases, EBV is detected by EBER-ISH (Epstein-Barr encoded RNAs–*in situ* hybridization) inside large B-immunoblasts morphologically similar to Reed-Sternberg cells (RS-cells) (42, 43). Additionally, monoclonal rearrangement of the immunoglobulin gene (IgVH) is seen in up to one third of AITL (44). Therefore, some authors were able to establish an association between high-tissue EBV viral load, the occurrence of B-monoclonality, and the development of diffuse large B-cell lymphoma (DLBCL), which can develop concomitantly or in a late evolutionary phase of AITL (4, 31, 45). It is speculated that EBV B-cell infection results from the characteristic immune dysregulation observed in AITL, however EBV may be able to modulate disease progression as well as the development of truly B-cell malignancies, such as DLBCL or plasmablastic lymphoma (PL).

Concerning to molecular aspects, AITL is characterized by the presence of recurrent mutations involving the *RhoA* gene and epigenetic regulatory genes, implicated in the processes of DNA-methylation, histone-deacetylation, and regulation of nuclear chromatin remodeling, such as *IDH-2*, *DNMT3A* and *TET-2* (3). The *RhoA* gene, located on chromosome 3, encodes a small GTPase that regulates cell migration, intracellular signaling, proliferation and survival. It also participates in the conformation of the cytoskeleton, signaling of the T-cell receptor (TCR) pathway, and plays a central role in the ontogenesis of T-lymphocytes (46–49). In its active state, the RhoA protein binds to guanine-triphosphate (GTP) and in its inactive state it binds to guanine- diphosphate (GDP). Mutant RhoA protein (RhoA-mut) has compromised GTP binding, which leads to alterations in the *RhoA* signaling pathway and impairment of its biological functions (24, 50). The *RhoA* G17V mutation, associated with loss of GTPase function, is recurrently found in up to 60-70% of AITL cases, and is currently considered a diagnostic biomarker for nMTCL-TFH-phenotype (24, 25, 50–52). Recent studies using animal models point to a clear relationship between the *RhoA* G17V mutation and TFH-cell differentiation, establishing a pathogenic link between this molecular alteration and AITL development (49, 53, 54). Based on these findings, it is postulated that the *RhoA* G17V mutation is a driver event for the development of nMTCL-TFH-phenotype, although it can still be found in neoplasms of different histogenesis, such as Burkitt’s lymphoma (BL), adult T-cell leukemia/lymphoma (ATLL) and gastric adenocarcinomas (24, 50, 55–57).

*TET-2* mutations (*Ten-eleven translocation 2*) were originally described in myeloid malignancies, such as myelodysplastic syndromes (MDS), chronic myelomonocytic leukemias (CMML), and acute myeloid leukemias (AML). However, later, these mutations have been identified recurrently in MTCL, particularly in those with TFH-phenotype (22, 58–60). Loss-of-function mutations on *TET-2* gene occur in up to 80% of AITL. This gene encodes an oxyglutarate-oxygenase that catalyzes the oxidation of DNA 5-methylcytosine (5-mC) to 5-hydroxy-methylcytosine (5-hmC) (61, 62). Experimental studies demonstrated that *TET-2*-mutant mices were prone to developing CMML and nMTCL-TFH-phenotype. These same studies associate suppression of the *TET-2* gene function with upregulation of the BCL-6 transcription factor and selection for differentiation of näive CD4+ T-cells for mature TFH-cells (21, 63, 64). Although *TET-2* mutations are considered secondary events in nMTCL-TFH-phenotype, different studies demonstrate an association of *TET-2* and *RhoA* G17V mutations in these neoplasms, suggesting a biological cooperation between both mutations to promote AITL development (49, 65). Recently, our research group demonstrated a high-rate of co-occurrence between *TET-2* and *RhoA* mutations in Brazilian patients with non-anaplastic nMTCL, confirming the data found in previous experimental studies (51, 52). In our population, composed of 59 patients with nMTCL, the *RhoA*-mut/*TET-2*-mut association was demonstrated in 42.8% of cases with nMTCL-TFH-phenotype, also being associated with a high-tumor volume represented by bulky disease ≥ 7 cm and decreased overall response rates (ORR) to primary treatment based on anthracyclines (51).

Up to 30-40% of AITL have a mutation in the *IDH-2* gene (*isocitrate dehydrogenase 2*) (23, 66). Physiologically, IDH enzymes catalyze the conversion of isocitric acid to 2-alpha-ketoglutarate (2-KG). Mutant IDH-2 enzymes will contribute to the malignant phenotype through the production of the aberrant metabolite 2-hydroxy-glutarate (2-HG) (23, 67). 2-HG inhibits the activity of TET family proteins and histone deacetylases, resulting in the production of 5-hmC and subsequent repression of tumor suppressor genes, resulting in the promotion of the malignant phenotype (68). Although it occurs recurrently in other neoplasms, such as AML and high-grade gliomas, *IDH-2* mutations, when present in MTCL, are practically exclusive to AITL and are usually restricted to the arginine residue 172 (R172K and R172S) (66, 69). Unlike cases of AML, where *IDH-2* and *TET-2* mutations are mutually exclusive and associated with adverse prognosis, in AITL they are usually seen concurrently, which suggests a cooperative effect between both mutations for expansion and differentiation of malignant TFH-cells (66).

Many other loss-of-function mutations, such as those involving the *DNMT3A* (*DNA methyl-transferase 3A*) gene, found in 10-40% of AITL, and mutations in genes associated with the TCR signaling pathway, such as *PLCG1*, *CD28*, *VAV1* and *FYN*, found in up to 50% of AITL also contribute to the development of the malignant phenotype in nMTCL-TFH-phenotype, although the latter are also frequently observed in PTCL, NOS and ATLL cases (25, 66, 70, 71).

**Figure 1:**
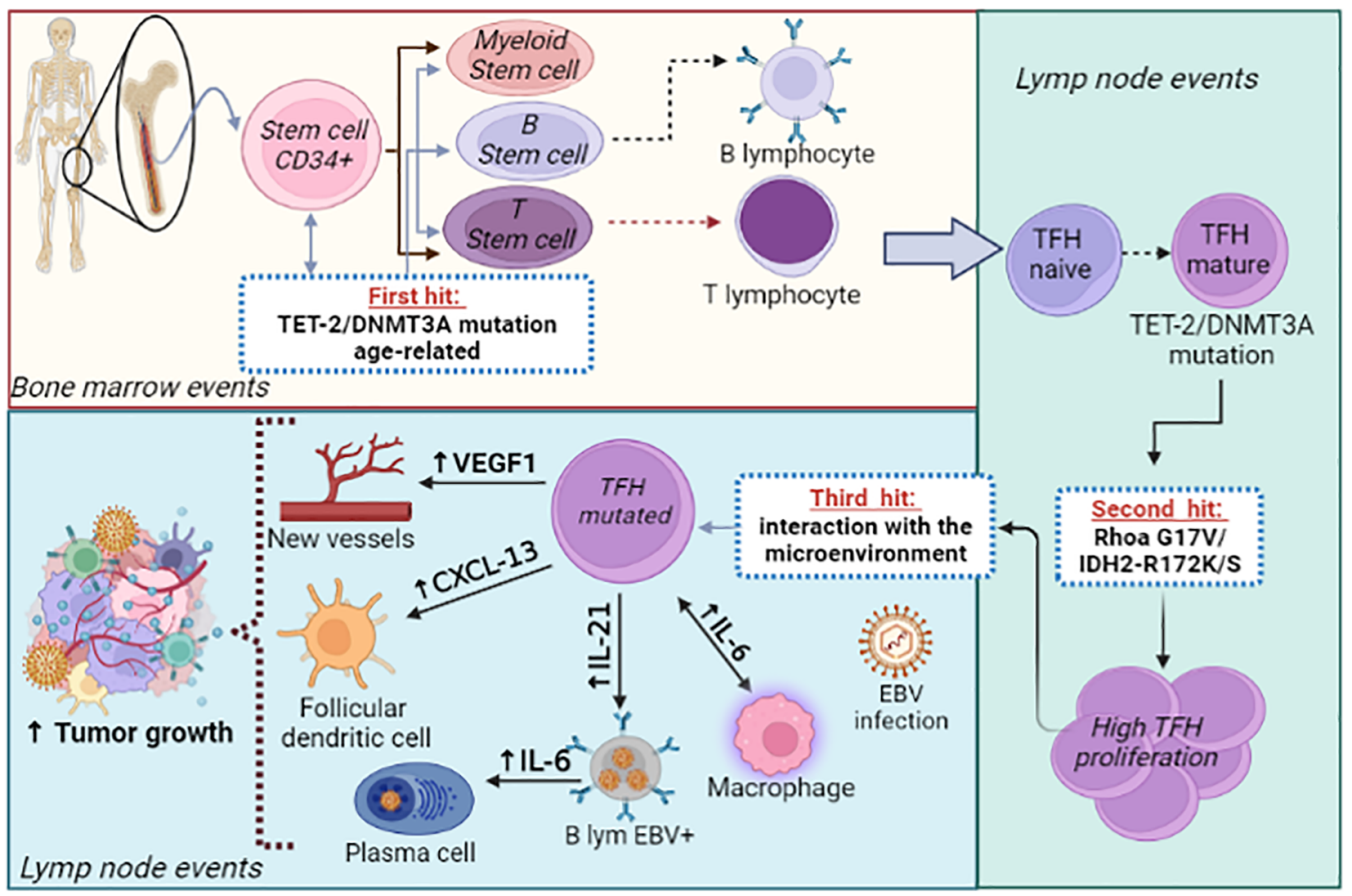
Integrative biologic model for AITL pathogenesis. AITL development follows a “*multi-step*” process. The initiating event of neoplasia (“*first-hit*”) involves age-related mutations in epigenetic regulatory genes (*TET-2* and/or *DNMT3A*) that compromise the pluripotent hematopoietic stem cells-CD34+ with propagation to myeloid precursor cells, B- and T-lymphoid cells. Subsequently, the naïve T-CD4+ cell matures to TFH-cell in the germinal center and experiences specific-disease (“driver”) mutations, such as the *RhoA* G17V and/or *IDH-2* R172 K/S (“*second-hit*”). Finally, mature and mutated TFH-cells expand in the germinal center from the interaction with several elements of the tumor immune microenvironment (FDC, macrophages, B-cells, endothelial-cells and EBV), which provide an environment marked by immune-dysregulation and pro-inflammatory activity, highly permissive for the propagation and dissemination of the neoplasm (“*third -hit*”).

Therefore, we conclude that the tumorigenesis of AITL and other nMTCL-THF-phenotype is a complex process, involving multiple steps of mutational phenomena associated with epigenetic regulation, leading to differentiation and expansion of malignant TFH-cells. These clonal TFH-cells, in turn, interact with a defective immune microenvironment that is permissive to the development of the neoplasm. In the meantime, self-reactivity phenomena and the EBV oncogenic activity, which proliferates in an immunodeficient microenvironment, favor tumor propagation and generate the classic immunodysplastic findings, typical of this group of lymphomas. Currently, it is speculated that age-related loss-of-function mutations in the *TET-2* and *DNMT3A* genes, which occur at an early-stage of hematopoietic development, constitute the initial events that promote the tumor (*first hit*), since they are found in clonal TFH-cells, in B-cells and in CD34+ myeloid precursors. Subsequently, the occurrence of specific-mutations, such as *RhoA* G17V and *IDH-2* R172K/S, observed exclusively in TFH-cells, lead to a second hit that modulates the differentiation and proliferation of clonal TFH-cells, orchestrating the development of AITL (32). It is now known that AITL, CMML and AITL-derived DLBCL are neoplasms that share common age-related ancestral mutations (*TET-2* and *DNMT3A*). Following this multi-step process, specific mutations such as *RhoA* G17V/*IDH-2*, *NPM-1* and *NOTCH-1* will predispose to the emergence and expansion of malignant clones of AITL, CMML and DLBCL, respectively, all of them considered as correlated hematopoietic neoplasms (46, 72–74). **Figure 1** demonstrates the previously summarized integrative pathogenic model of AITL.

## 4 Histopathological diagnosis

As for other MTCL, excisional lymph node biopsy is the preferred procedure for establishing the diagnosis of AITL and other nMTCL-TFH-phenotype. Although incisional or core needle biopsies can establish this diagnosis, a complete architectural evaluation of the affected lymphoid tissue in the AITL is usually required, with a detailed analysis of the tumor compartment and the exuberant immune microenvironment, which many times can only be achieved with histopathological analysis of the entire lymph node. Additionally, large amounts of tumor tissue are needed to carry out a broad immunohistochemical panel, *in situ* hybridization (ISH) for EBV and complementary molecular tests, such as T-cell clonality assays by PCR and mutational profiling involving the *RhoA* gene and other epigenetic regulators by Sanger sequencing or next-generation sequencing (NGS) techniques.

Classically, AITL can be characterized by partial lymph node involvement, or more commonly by diffuse nodal infiltration with complete rupture of the normal tissue architecture, as well as capsular and perinodal infiltration frequently sparing the peripheral sinus, and absence of residual B-cell lymphoid follicles (2, 75). According to Attygalle et al., three distinct architectural patterns can be observed in the AITL. Pattern I, or AITL with hyperplastic follicles, found in 15% of cases, exhibits partially preserved nodal architecture and hyperplastic lymphoid follicles with poorly developed mantle zones. In pattern II, or AITL with depleted follicles, observed in 25% of cases, occasionally depleted follicles are present and the FDC meshwork is unchanged or minimally expanded (20). In pattern III, the so-called classic AITL without follicles, which occurs in 60% of cases, there is complete loss of the architecture of the affected lymph node, capsular and perinodal infiltration sparing the peripheral sinus, and absence of residual B-cell lymphoid follicles (20). Different histopathological patterns have been documented in consecutive biopsies and seem to represent progressive stages of the neoplasia. This purely reflects a morphological evolution and is not associated with clinical progression, since patients with pattern I usually present with symptomatic and advanced-stage disease (20, 76).

The histopathological findings of classic AITL (pattern III) include: (I) diffuse and polymorphic infiltrate, composed of a variable proportion of tumor cells with TFH-phenotype, interspersed with small reactive lymphocytes, histiocytes or epithelioid cells, large B-immunoblasts, eosinophils and plasma cells; (II) prominent proliferation of high endothelium venules (HEV) in an arborescent pattern; (III) expansion of the FDC meshwork, often accompanying the proliferated vessels; and (IV) expansion of large immunoblasts of B-lymphoid phenotype in the paracortical area, with frequent Reed-Sternberg-like (RS) morphology and expressing small-RNAs encoded by the EBV (EBERs) (2, 11, 20, 77).

In AITL, the neoplastic cells with TFH-phenotype are usually small to medium-sized and have mild atypia. Its cytoplasm is abundantly clear (“*clear cells*”), with a tendency to form small aggregates surrounding HEV (78). Expansion of B-immunoblasts is usually present in the paracortex and may mimic reactive immunoblastic hyperplasia or cHL by its RS-like morphology (79). Tissue plasmacytosis can represent an exuberant finding in several cases, obscuring the tumor cells (2, 77). The amount and distribution of neoplastic cells and the different components of the tumor microenvironment differ widely between cases; however, usually clear tumor cells comprise the minority (about 30%) of the nucleated elements in AITL (2).

In the immunohistochemical study, the lymph nodes involved by AITL show expansion of the paracortex by a diffuse infiltrate of CD4+ T-cells. B-cell lymphoid areas are usually reduced, although large speckled B-immunoblasts, often CD30+ and EBV+ are found among tumor cells (2, 77). Usually, neoplastic cells show expression of pan-T markers, such as CD2+, CD3+ and CD5+, although loss of expression of these antigens, particularly surface CD3 (sCD3) and CD7 can occur in up to 50% of cases (27, 80). By flow cytometry, loss of sCD3 is commonly observed and can be an important diagnostic clue (81, 82). Tumor cells are CD4+, although the CD4/CD8 ratio is usually preserved in lymph nodes involved by AITL (83). A portion of AITL cases (20-30%) may exhibit partial expression of the Ki-1 marker (CD30) (2, 77).

Being a neoplasm of TFH-origin, AITL displays multiple TFH-related antigens, including PD-1/CD279 (*programmed death-1*), CD10 (a metalloendopeptidase called *common acute lymphoblastic leukemia antigen*/CALLA), BCL-6 (*B-cell lymphoma 6 protein*), CXCL-13 (*C-X-C motif chemokine ligand 13*), ICOS (*inducible T-cell costimulator*), SAP (*signaling lymphocyte activation molecule [SLAM]-associated protein*) and CXCR-5 (*C-X-C motif chemokine receptor 5*) (11, 35, 36, 77, 84, 85). It should be noted that these markers are not specific, so it is recommended that at least two of them, or preferably three, be expressed by the neoplastic cell to define that a nMTCL has a TFH-phenotype (2, 77). Among the TFH-markers, PD-1/CD279 and ICOS are reported to be more sensitive, while CXCL-13 and CD10 are less sensitive but more specific (86).

In the immunohistochemical study, the exuberant vascular proliferation characteristically observed in AITL may be better evidenced by the endothelial markers CD31 and CD34, as well as the expanded FDC meshwork, evidenced by staining for CD21, CD23 and CD35 antigens. CD138 staining will help to show exuberant plasmacytosis, often seen in AITL. Although plasma cells are usually polyclonal, truly clonal plasma cell proliferations have rarely been described (87, 88).

Polyclonal or truly monoclonal EBV+ lymphoid B-cell proliferation will often be seen in AITL and appear to result from immunoderegulation mediated by tumor TFH-cells. Different studies demonstrate that up to 80% of AITL cases may contain a variable number of EBV+ B-cells, ranging from isolated foci of paracortical immunoblasts to true EBV-associated mature B-cell lymphoproliferation, such as EBV+DLBCL, which can be observed both in the initial diagnosis of AITL (synchronous neoplasms) or during disease progression (89–91).

**Figure 2:**
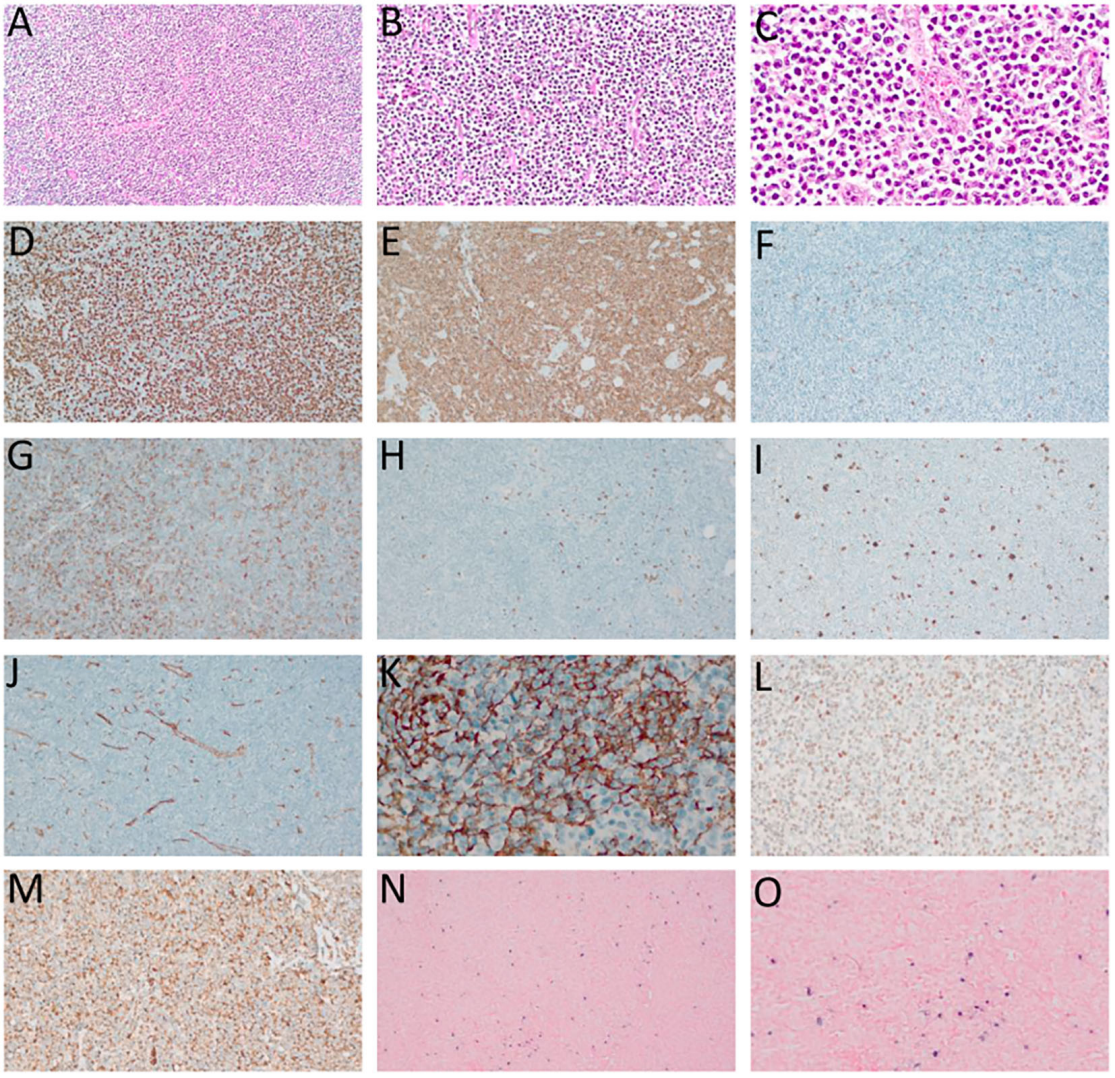
Angioimmunoblastic T-cell lymphoma (AITL). **(A–C)** – Hematoxylin-eosin (HE), optical microscopy, magnifications of 10 x **(A)**, 20 x **(B)** and 40 x **(C)**. Proliferation of small/medium sized atypical lymphoid cells diffusely infiltrating the lymph node with marked vascular proliferation. **(D)** high-rate of cell proliferation index - positive Ki-67 in more than 80% of the nuclei of atypical lymphoid cells. **(E)** Strong and diffuse labeling for the pan-T CD3 antigen. **(F)** Rare large CD20-positive cells in the paracortical region (“immunoblasts”). **(G)** CD4-positive in most neoplastic cells. **(H)** CD8-negative in neoplastic cells and positive in rare small reactive lymphocytes. **(I)** CD30-positive in large, expanded cells in the paracortical region with RS-like morphology. **(J)** Exuberant vascular proliferation with high endothelial venules (VEA) enhanced by CD34. **(K)** Expanded follicular dendritic cell (FDC) meshwork, labeling for CD21. **(L)** BCL6 positive and **(M)** CD10 positive, both markers of TFH origin. **(N, O)** *In situ* hybridization (ISH) for EBV, positivity for EBERs (Epstein-Barr small encoded-RNAs), staining in black, “speckled” pattern, revealing EBV staining in RS-like immunoblasts in the paracortex. **(D–F, H, L–N)** – Optical microscopy, 10 x magnification. **(I, J, O)** – Optical microscopy, 20 x magnification; **(K)** – Optical microscopy, 40 x magnification.

**Figure 3:**
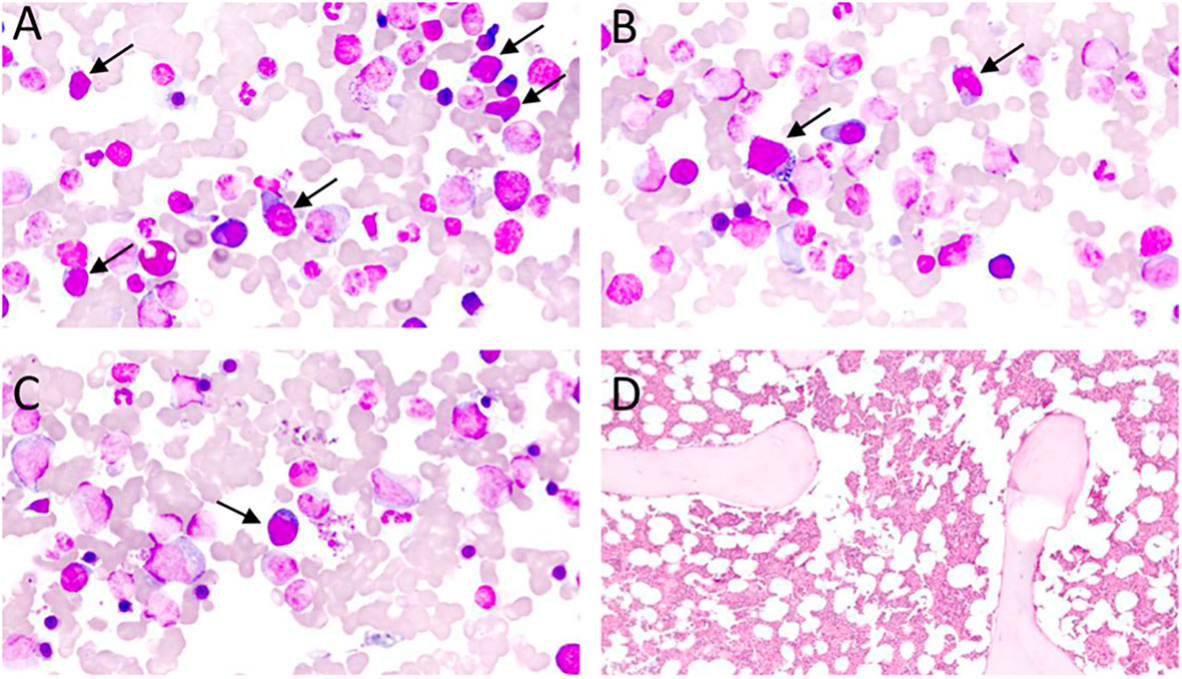
**(A–C)** – Bone marrow aspirate, Leishman staining, optic microscopy, 70 x magnification: subtle bone marrow infiltration by atypical lymphoid cells (black arrows) in an AITL case. Atypical lymphoid cells are small/medium-sizes, have loose chromatin, cytoplasm with pronounced basophilia, and some forms have microvacuolation, making up about 10% of the nucleated elements in the bone marrow specimen. **(D)** – Bone marrow biopsy, Hematoxylin-Eosin (HE), optical microscopy, 10 x magnification. Usually, lymphomatous infiltration of the bone marrow in AITL is subtle and difficult to characterize in HE, requiring immunohistochemical staining for TFH-associated antigens for better revelation of neoplastic cells.

Skin and bone marrow are also commonly affected in AITL. In the skin, histological findings are usually subtle, including perivascular and periadnexal lymphoid infiltrates with mild atypia, conditions that are difficult to distinguish from inflammatory dermatoses. Immunohistochemistry for TFH-markers and T-cell clonality assays (PCR) can be very useful to distinguish reactive paraneoplastic skin rash from a cutaneous tumor infiltration by AITL (77). In the bone marrow, neoplastic infiltration by AITL can be characterized by nodular lymphoid infiltrates of paratrabecular or interstitial distribution, commonly accompanied by trilineage dysplasia, fibrosis, and plasmacytosis. TFH-associated markers usually have a lower yield in the bone marrow than in the lymph nodes, possibly due to impaired antigenic recovery influenced by sample decalcification (2, 77). **Figures 2**, **3** summarize the main histological and immunohistochemical findings observed in classic cases of AITL in lymph node and bone marrow.

AITL shares many histopathological findings with other nMTCL-TFH-phenotype, so-called AITL-related disorders, due to the overlap of phenotypic and molecular aspects. In follicular-type nTFHL (nTFHL-F), the affected lymph nodes present a predominantly nodular growth pattern, similar to that observed in follicular lymphoma. However, the exuberant inflammatory background observed in AITL, as well as vascular proliferation with HEV and expansion of the FDC meshwork are not observed (2, 11, 12, 77).

## 5 Clinical and laboratory features

Although up to 20-25% of AITL cases may have an indolent and fluctuating course, the disease usually has a clinical spectrum marked by high aggressiveness (1, 10, 28, 80). Clinically, the disease affects elderly men, with a median age around the sixth decade of life, as generalized lymphadenopathy associated with manifestations of autoimmunity and immunodeficiency (31, 80). The clinical manifestations resulting from hyper-inflammatory reaction, autoimmunity, and immunodeficiency, causing greater susceptibility to infections, constitute the so-called “*angioimmunoblastic lymphoma syndrome*” or “*immunodysplastic syndrome*” (3, 31). Prototypically, AITL manifests as an acute or subacute systemic disease after administration of drugs or viral infections, establishing a wide differential diagnosis with pharmacodermias, collagenosis and exanthematic viral diseases, which has led some authors to recognize this disorder as “*the many-faced lymphoma*” (3).

The majority of AITL patients have advanced-stage disease (Ann Arbor III or IV), although rare cases of early-disease (I or II) have been reported and constitute less than 10% of clinical presentations (3, 10, 31). Although considered within the scope of predominantly nodal MTCL, extranodal involvement is not infrequent in AITL. Bone marrow infiltration has been reported in up to 70% of cases, as well as involvement of other extranodal sites, such as the lungs and the gastrointestinal tract (3, 31). Peripheral blood involvement by small circulating clones, particularly identified by flow cytometry, usually with sCD3-/CD4+/CD10+ phenotype can be identified (81).

**Figure 4:**
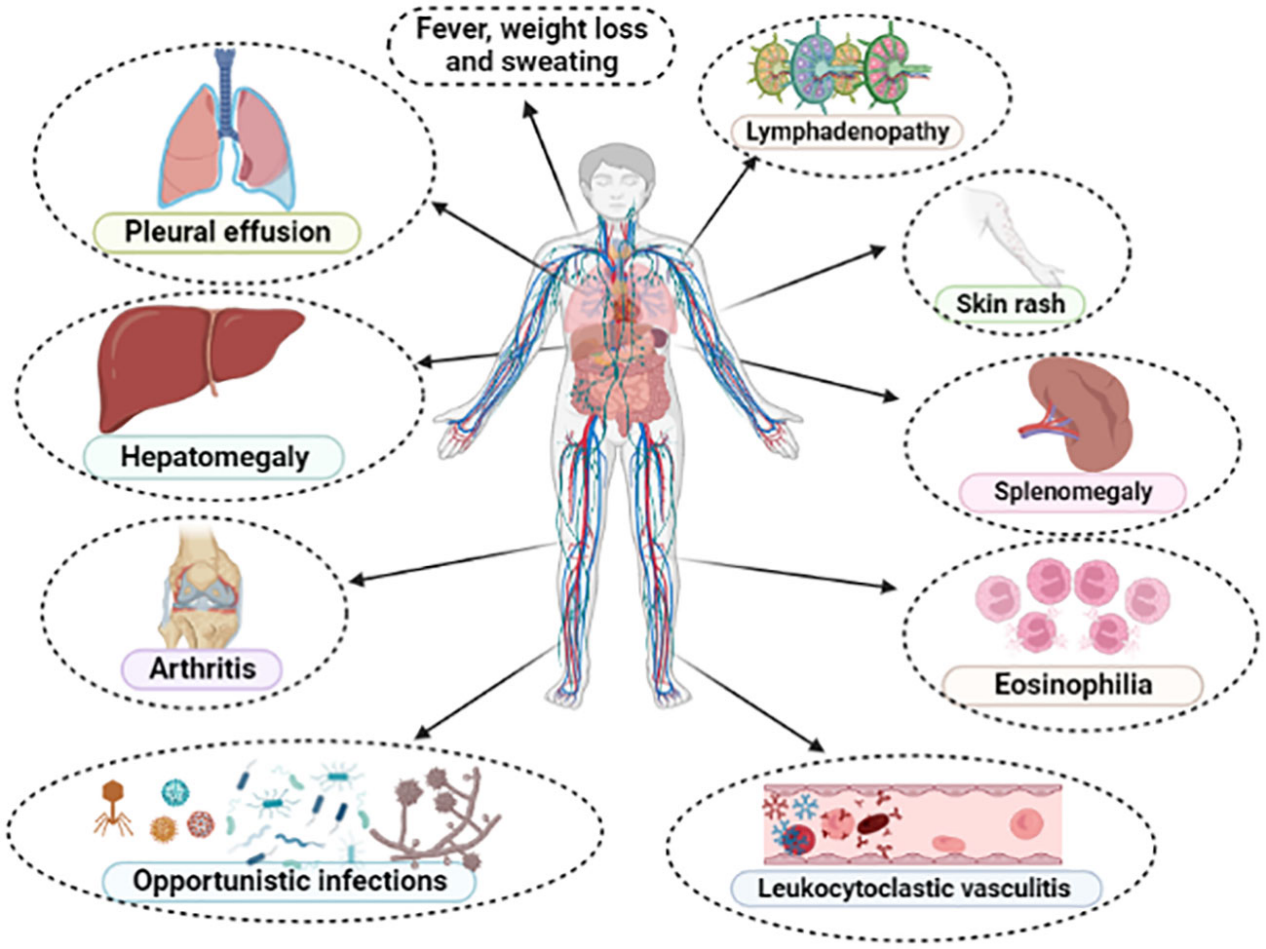
Main clinical and laboratory findings presented in typical cases of angioimmunoblastic T-cell lymphoma (AITL). All these manifestations compose the so-called “*angioimmunoblastic lymphoma syndrome*” or “*immunodysplastic syndrome*”, establishing differential diagnosis with drug-reactions, collagenoses and exanthematic viral infections.

Characteristically, AITL patients present with constitutional symptoms (B-symptoms), such as fever, weight loss, and night sweats. Lymphadenopathy is usually generalized and small (< 1-3 cm), although our group observed a high occurrence of bulky disease ≥ 7 cm in South American patients (29, 31). Hepato-splenomegaly is frequently observed, as well as skin rash and pruritus, the latter occurring in up to 50% of cases, and may be reactive or correspond to frank cutaneous tumor infiltration by the TFH-clone (3, 31, 77). The typical immunoderegulation of AITL predisposes to immunodeficiency and greater susceptibility to bacterial, fungal, and opportunistic infections (3, 31, 89). Manifestations associated with increased vascular permeability, resulting from the hypersecretion of VEGF-1 (92, 93), such as peripheral edema, pleural effusion and ascites are observed in up to 30% of cases (3, 30). Autoimmune phenomena such as leukocytoclastic vasculitis, thyroiditis, arthralgia and/or arthritis are recurrently observed (3, 31). Laboratory findings include autoimmune hemolytic anemia (Coombs positive), polyclonal hypergammaglobulinemia, elevated erythrocyte sedimentation rate (ESR), eosinophilia, lymphopenia, and thrombocytopenia, although some patients may present with atypical lymphocytosis with circulating hyper basophilic lymphoid cells (3, 31, 94, 95). Serum markers of autoimmunity, such as antinuclear antibodies (ANA), rheumatoid factor, anti-smooth muscle antibodies, circulating immune complexes, cryoglobulins and cryoagglutinins are described in up to 20-30% of cases (3, 30, 31). **Figure 4** summarizes the spectrum of clinical and laboratory manifestations recurrently observed in AITL and related disorders.

In opposition to other MTCL, such as ALK1-positive anaplastic large-cell lymphoma (ALK1+ ALCL), associated with the t(2;5)(p23;q35), and T-cell prolymphocytic leukemia (T-PLL), associated with inv (14)(q11;q32)/t(14;14)(q11;q32), AITL does not present a characteristic or recurrent chromosomal abnormality. However, cases of nTFHL-F are recurrently associated with t(5;9)(q33;q22) and consequent *splenic tyrosine kinase* (SYK) upregulation (96). In AITL, more than 90% of cases demonstrate clonal aberrations, the most common being trisomy of chromosomes +3, +5, +21, +X and deletion of chromosome 6q-. Inactivation of the *TP53*, associated with the abnormalities del(17p-)/-17 and complex karyotype are rare clonogenic alterations in AITL associated with poor outcomes (94, 97). T-cell and B-cell clonality assays using PCR techniques to assess clonal rearrangements in the TCR and immunoglobulin heavy-chain (IgVH) gene occur in 80-100% and up to 50% of AITL, respectively (31, 98).

Recent studies involving NGS techniques have demonstrated variable proportions of recurrent mutations involving the *RhoA* GTPase, epigenetic regulatory genes (*IDH-2*, *TET-2* and *DNMT3A*) and genes involved in the TCR signaling pathway (*PLCγ*, *CD28*, *FYN* and *VAV-1*) in patients with AITL (99, 100). *RhoA* mutations, particularly G17V have been reported in 60-70% of cases, *TET-2* in 47-83%, *DNMT3A* in 20-30% and *IDH-2* R172K/S in 20-45% of cases. Mutations in genes associated with the TCR signaling pathway are rarer, occurring in 14% of cases, 9-11%, 3-4% and 5%, respectively, for *PLCγ*, *CD28*, *FYN* and *VAV-1* genes (13, 23–25, 50, 60, 71, 101).

## 6 Outcomes and prognostic factors:

Although it is a tumor with a highly fatal course, 32-41% and 18-38% of AITL patients will survive after 5 years of diagnosis and will remain event-free during this period, respectively, according to data from the *International T-cell Lymphoma Project* (ITCLP), as well as to other large collaborative studies (10, 27, 30, 80, 102). In the last two decades, since the initial report by the *ITCLP* in 2008, the outcomes of patients with AITL have not presented significant increments, despite the great advances in its biological knowledge. Newly published data involving a subgroup of 282 AITL cases recorded in the *ITCLP* from 2006 to 2018 demonstrate OS and progression-free survival (PFS) at 5 years of 44% and 32%, respectively (28). In parallel, this same study pointed to progression of disease within 24 months from diagnosis (POD-24) as a powerful predictor of prognosis in patients with AITL, with an estimated 5-year OS of 63% versus only 6% for patients without POD-24 and with POD-24, respectively, p<0.001 (28). Data from our research group, involving 124- Brazilian cases with nMTCL diagnosed and treated at the University of São Paulo, from 2000 to 2019, with 10.4% (13/124) of AITL among all cases, revealed similar outcomes. In our cohort, the estimated 2-year OS and PFS for South American patients with AITL was 53.8% and 45.5%, respectively, corroborating the poor clinical outcomes associated with this tumor, as previously reported in other studies conducted in Europe and North America (29).

Prognostic scores, such as the *International Prognostic Index* (IPI) and the *Prognostic Index for Peripheral T-cell Lymphoma Unspecified* (PTCL-U score), the latter including the variables age ≥ 60 years, LDH ≥ normal value, performance status according to the scale *Eastern Cooperative Oncology Group* (ECOG) ≥ 2 and histopathological bone marrow involvement by NHL, in addition to reduced platelet count, have been able to predict prognosis in AITL patients (10, 30, 80). Recently, Advani et al. reported a new risk-score, denominated *AITL score*, contemplating the independent variables age ≥ 60 years, ECOG performance status ≥ 2, high C-reactive protein levels, and β2-microglobulin ≥ normal value, as having a high ability to discriminate clinical outcomes in cases of AITL (28). According to this risk-score, patients categorized as low-, intermediate-, and high-risk had 5-year OS estimates of 63%, 54%, and 21%, respectively (28). Similarly, Hong et al., retrospectively analyzing 115 patients with AITL, identified as independent predictors associated with poor survival the histological involvement of bone marrow, involvement of > 1 extranodal site by NHL and performance status > 1. In this prognostic score, patients were categorized into three groups, with 5-year OS estimates of 86.9%, 46.3% and 16.2%, respectively, p<0.0001. According to the authors, this score showed better predictive discrimination to determine survival for AITL patients when compared to the IPI and PTCL-U scores (103).

Laboratory and molecular-genetic biomarkers have emerged in recent decades as methods for predicting prognosis in several hematological malignancies, including AITL. Therefore, high peripheral monocyte count at diagnosis, thrombocytopenia, high-levels of immunoglobulin A (IgA), serum albumin < 35 g/L, high-levels of β2-microglobulin, reduced lymphocyte/monocyte ratio, presence of *TET-2* mutations in diagnostic biopsies, the *RhoA* G17V mutation, high tumor mutational burden (TMB) involving a gene panel composed by the *RhoA* GTPase and epigenetic regulators genes (*IDH-2*, *TET-2* and *DNMT3A*), gene signature pattern related to monocytes and *TP53*, as well as tumor status for EBV assessed by EBER-ISH in lymph nodes (EBER-negative status) adversely impacted the survival of AITL patients according to data published by different research groups in the last ten years (51, 52, 104–109).

## 7 Treatment

Up-front therapy for AITL and other nMTCL-TFH-phenotype may be focused on different objectives, from curative proposals to strategies with a merely palliative aim. Young patients aged less than 60-65 years, with good performance status (ECOG ≤ 2) and without significant comorbidities will experience multidrug therapy based on anthracyclic agents with curative intent, preferably followed by consolidation in first complete remission/partial response (CR/PR) with high-dose therapy (HDT) and autologous hematopoietic stem cell transplantation (ASCT). Although it is considered the gold-standard therapy outside the clinical trial scenario, this approach is still not able to promote high-rates of long-term sustained remission, being associated with an estimated 5-year OS around 30-40% (3, 10, 28, 110). On the other hand, elderly patients (≥ 60-65 years old) with poor clinical conditions and/or severe comorbidities (“frail patients”) will be treated with palliative intent, due to the high morbidity and mortality rates presented by this population when submitted to intensified therapeutic strategies. Therefore, this subgroup of patients may be approached with a combination of corticosteroids and low-dose chemotherapy, as well as the isolated use of steroids, calcineurin inhibitors (cyclosporine A), immunomodulators (lenalidomide), use of the immunoconjugate drug-monoclonal antibody anti-CD30 (brentuximab-vedotin/BV), gemcitabine-based monochemotherapy, or strategies focused on epigenetic regulation, such as the association of hypomethylating agents (HMAs) and histone deacetylase inhibitors (HDAi) (110, 111). At the same time, AITL elderly patients (≥ 60 years) without serious comorbidities and presenting good performance status, categorized as an “intermediate-age” population (between 60 – 75 to 80 years), may benefit from a full course of cytotoxic chemotherapy (e.g. CHOP or BV-CHP regimens), even if ineligible for consolidation with ASCT.

As observed in other nMTCL, patients with AITL exhibit high-rates of resistance to anthracyclic-based chemotherapy, primarily centered on the CHOP regimen (cyclophosphamide, doxorubicin, vincristine, and prednisone) (3, 10, 29, 110, 111). Currently, it is known that this is due to the high-concentration of P-glycoprotein (Pgp) present in nMTCL tumor cells, which confers a multidrug resistance (MDR) phenotype. By generating an efflux pump mechanism for chemotherapeutic agents, notably against anthracyclines and vinca alkaloids, nMTCL tumor cells block the cytoplasmic internalization of these drugs, thus preventing their antineoplastic effect (112–114). Although CHOP-like regimens are still considered the “standard of care” for AITL patients treated with curative intent, the responses obtained with this strategy are considered poor, with overall response rates (ORR) around 70-79% and complete response (CR) around 35-39% (10, 115, 116). Similarly, long-term outcomes have been highly unsatisfactory for AITL patients treated with anthracycline-based regimens as reported by different groups, with 5-year PFS estimates of 18%, 13%, and 20% according to the *ITCLP*, *British Columbia Cancer Agency* (BCCA) group, and *Swedish National Lymphoma Registry*, respectively (10, 115, 116). This has led to several guidelines, including that of the *National Comprehensive Cancer Network* (NCCN), to recommend enlisting patients with AITL in clinical trials as preferred primary therapeutic strategy (117).

Due to the rarity of AITL, which represents only 2% of all NHL, the majority of studies that aim to analyze the effectiveness of different therapeutic strategies include AITL patients together with others subtypes of nMTCL, such as PTCL, NOS and ALCL, which makes it difficult to interpret the results. Therefore, most of the studies presented in the next paragraphs do not exclusively involve patients with AITL, although those with greater scientific robustness present results for patients with AITL in the form of subgroup analysis.

Aiming to improve the outcomes provided by the CHOP regimen in patients with nMTCL, several strategies have been conducted over the last two decades. Among the main ones, we highlight the addition of other drugs to the CHOP protocol, such as etoposide (CHOEP regimen), the anti-CD52 monoclonal antibody alemtuzumab (CHOP plus alemtuzumab), the proteasome inhibitor bortezomib (V-CAP regimen), association with epigenetic modulators (CHOP plus 5-azacitidin or romidepsin), in addition to more intensified multi-drug regimens such as ICE/ABVD (ifosfamide, carboplatin, etoposide/adriamycin, bleomycin, vinblastine and dacarbazine), PEGS (cisplatin, etoposide, gemcitabine and solumedrol), ACVBP (doxorubicin, cyclophosphamide, vindesine, bleomycin and prednisone), among many others. Overall, although some of these combinations promoted higher overall response rates, toxicity was notably increased and there was no unequivocal benefit in overall survival (29, 116, 118–124).

The first strategy used to intensify the CHOP regimen in the scenario of nMTCL was based on the addition of etoposide at a dose of 100 mg/sqm I.V. on the first three days of the regimen with dosing intervals every 14 or 21 days (CHOEP-14/21). Although a study conducted by the *German High-Grade NHL Study Group* involving 289 patients with nMTCL demonstrated a 3-year event-free survival (EFS) benefit for patients with ALK1+ ALCL, specifically for those younger than 60 years and with normal levels of LDH (p=0.003), this benefit was only marginal (p=0.057) for non-ALK1+ subtypes, including AITL patients. In this same cohort, elderly patients (aged > 60 years) experienced prohibitive toxicity with the addition of etoposide to the CHOP regimen. Furthermore, even in the group of younger patients, CHOP plus etoposide did not demonstrate an unequivocal benefit in terms of overall survival for any of the nMTCL histological subtypes (118). Similar data from the *Swedish National Lymphoma Registry*, with 252 cases of nMTCL treated over a period of more than 10 years showed that the addition of etoposide to CHOP caused higher ORR and increased PFS in patients younger than 60 years (HR 0.49, p=0.008), without, however, leading to greater overall survival (116).

In opposition to previous studies, a recent publication involving 1427 patients enrolled in the *Netherlands Cancer Registry* between 1989 and 2018 demonstrated for the first time a 5-year OS advantage for the CHOEP versus CHOP regimen (64% and 44%, respectively, p<0.01). However, after adjustment for histological subtype, this benefit was maintained only for patients with ALK1+ ALCL, with a 6.3-fold increased risk of mortality among those treated with CHOP. In this analysis, patients < 65 years with ALK1-negative ALCL, AITL, and PTCL, NOS subtypes did not have increased OS with addition of etoposide to CHOP (125). In 2021, Kim J et al., conducted a meta-analysis involving 34 cohorts from 28 studies and a pool of 1424 patients with nMTCL. This meta-analysis demonstrated OS benefit for the CHOEP regimen versus the CHOP regimen (126). However, in contrast, another recent meta-analysis involving 1560 patients with nMTCL from five retrospective and prospective studies was unable to demonstrate differences in CR, PR, and survival between CHOP and CHOEP regimens. As expected, in this meta-analysis patients with nMTCL treated with CHOEP-21 had higher hematologic toxicity, including higher rates of anemia and thrombocytopenia (127).

In a recent retrospective analysis published by our group, involving 124-Brazilian patients with nMTCL, treated under real-life conditions between 2000 and 2019, we demonstrated similar ORR between cases treated with CHOP-21 and CHOEP-21 (76.6% and 65.9%, respectively, p=0.259). Although CHOEP-21 was associated with a lower rate of primary refractoriness (4.5% vs 21.2%, p=0.018), the combination of CHOP plus etoposide was correlated with higher rates of delay between chemotherapy cycles (p=0.0004), definitive interruption of treatment (p=0.003) and toxicities, including severe neutropenia (p=0.001), febrile neutropenia (p=0.003) and severe thrombocytopenia (p=0.0007). Therefore, we assume that the high toxicities of the CHOEP-21 regimen, when applied to a population of particularly frail patients with nMCTL, may explain the absence of OS and PFS benefit, as demonstrated in our study (29). In summary, even today, the addition of etoposide to the CHOP regimen presents conflicting results regarding its potential benefit in the scenario of nMTCL, particularly in non-ALK1+ ALCL cases, such as AITL and correlated neoplasms with TFH-phenotype. Specifically for the population with AITL, no study was able to demonstrate an OS advantage for the CHOEP regimen compared to CHOP.

Based on the biological rationale associated to VEGF-1 overexpression, with consequent increase in tumor vascular density, as well as the immune dysfunction commonly observed in AITL, the use of agents with anti-angiogenic and immunomodulatory properties have been tested as potential new therapeutic weapons in this lymphoma. In this regard, the effects of adding the humanized anti-VEGF1 monoclonal antibody bevacizumab to the CHOP regimen followed by maintenance with monodrug bevacizumab was tested in a phase II study involving 46 patients with nMTCL. Despite promoting high ORR, the CHOP plus bevacizumab regimen failed to provide durable remissions and was associated with high-rates of toxicity, particularly myelotoxicity, and an increased risk of serious cardiovascular events (128). Following the same rationale, addition of the immunomodulatory and anti-angiogenic agent lenalidomide to the CHOP-21 regimen was recently tested in a phase II multicenter study including 80 treatment-naïve elderly AITL patients. In this study, the primary endpoint (complete metabolic response [CMR] at the end of treatment) was reached in only 41% of cases, being below the CMR rate of 55%, pre-specified as the adopted success rate. The 2-year PFS and OS were 42.1% and 59.2%, respectively, with non-negligible hematological toxicity, which resulted in discontinuation of therapy in 15% of cases. Interestingly, in this prospective study, with broad molecular characterization of the included cases, mutations in the *DNMT3A* gene were associated with shortened PFS and *IDH-2* mutations were associated with specific pathological findings, including FDC expansion, presence of clear neoplastic cells and bone marrow infiltration (129).

Due to the frequent interactions between TFH-tumor cells and CD20+ B-cells that make up the AITL tumor microenvironment, together with the fact that large CD20+ B-immunoblasts containing EBV are expanding in the paracortex of AITL lymph nodes, there is a rationale for the use of therapies that deplete B-lymphocytes, such as the anti-CD20 monoclonal antibody rituximab in this tumor. Based on this premise, Delfau-Larue et al. conducted a phase II study using 8 cycles of R-CHOP-21 in 25 patients newly diagnosed with AITL. With a CR rate of only 44% and an estimated 2-year PFS of 42%, this study did not demonstrate a clear benefit of adding rituximab to conventional anthracycline-based chemotherapy in patients with AITL (106). A recent publication, involving 335 AITL patients registered in the *Netherlands Cancer Registry* between 2014 and 2020, confirmed the findings previously cited by the French group. In the Dutch study, although addition of rituximab to CHOP-like regimens (CHOP-21 or CHOEP-21) improved ORR, there was no increase in OS or PFS with adoption of this strategy (130).

Expression of the CD30 antigen has been described in 43-90% of AITL cases, which opened a precedent for the use of the anti-CD30 drug-antibody immunoconjugate brentuximab vedotin (BV) in its primary treatment. In the phase III, multicenter, prospective Echelon-2 study, 452 patients with CD30+ nMTCL were up-front treated with BV-CHP regimen (Brentuximab-vedotin, cyclophosphamide, doxorubicin, and prednisone) versus CHOP for 6 to 8 cycles with a 21-day interval between administrations. We highlight the fact that more than 70% of the patients included in the study had ALCL, a universally CD30+ neoplasm associated with a high density of antigen expression. On the other hand, only 13% of this cohort involved patients with AITL. The BV-CHP group had substantially higher rates of complete response (p<0.01), as well as unequivocal benefit of OS and PFS, with a median PFS of 48 months versus 20 months for BV-CHP and CHOP, respectively, p=0.01. Furthermore, the rate of serious adverse events, including myelotoxicity and peripheral neuropathy, did not differ statistically significantly between both treatment arms. The data presented by Echelon-2 represented a paradigm shift in the primary therapy of CD30+ nMTCL, particularly for ALCL, where the BV-CHP regimen is currently considered the gold standard of care. However, we highlight the fact that in subgroup analysis, in opposition to what was evidenced for CD30+ PTCL, NOS and ALCL (ALK1+/ALK1-), patients with CD30+ AITL treated with the BV-CHP regimen had decreased PFS than those treated with CHOP (HR: 1.40, 95% CI 0.64-3.07) (131).

The discovery of recurrent mutations involving epigenetic regulatory genes in patients with AITL and in other nMTCL-TFH-phenotype opened the opportunity for the incorporation of epigenetic modifying drugs in the therapeutic arsenal of these lymphomas (132). Therefore, HMAs, particularly 5-azacytidine, and several HDAi, such as romidepsin, belinostat and vorinostat have been used in monotherapy or in combination for AITL therapy. Recent studies have shown promising results and relative safety for these drugs, both when used in the first-line setting or in R/R disease (120, 133–136).

In 2018, Lemmonier et al., published a report demonstrating the effectiveness of 5-azacytidine in inducing sustained responses in AITL. In this study, the authors used 5-azacytidine in monotherapy, applied subcutaneously in a retrospective series of 12 AITL patients treated with HMAs for concomitant myeloid neoplasia or in the setting of R/R disease. The ORR was 75%, with 50% CR and 25% PR. With a median follow-up of 27 months, the median OS and PFS were 21 months and 15 months, respectively. Due to sample limitations, the authors could not establish the impact of the mutational status of the *RhoA*, *TET-2*, *DNMT3A* and *IDH-2* genes in therapeutic response and survival (133). Subsequently, O’Connor et al. conducted a phase I study to assess the efficacy and safety of combining oral azacytidine with intravenous romidepsin in patients with advanced R/R lymphoid malignancies, with an emphasis on MTCL. This study determined the maximum tolerated dose of this association (300 mg of azacytidine P.O. on days 1 to 14 and romidepsin 14 mg/sqm I.V. on days 1, 8, 15 and 22, with an interval of 35 days between cycles) and revealed the efficacy and safety of the combination. The overall response and complete response rate were 73% and 55% in patients with MTCL, however the authors did not find association between response and level of demethylation or tumor mutational profile (135). In 2021, the same group published data from a phase II multicenter study testing the same association in 25 cases of R/R MTCL. In that study, ORR and CR were 61% and 48%, respectively. Interestingly, cases with TFH-phenotype were particularly susceptible to the combination of epigenetic modifiers, with ORR and CR of 80% and 67%, respectively. This regimen was shown to be safe and capable of inducing long-lasting responses in patients with R/R disease, with a median response duration of 20.3 months and median OS not reached. In a pioneering way, the authors were able to establish an association between response and greater mutational burden involving epigenetic regulatory genes (134).

Although the use of epigenetic modifiers has shown promising results in R/R disease, their use in the first-line setting, particularly for fit patients, is premature and has led to controversial results. In this sense, studies using the up-front CHOP regimen in association with epigenetic modifiers have had their results recently published. In 2020, Jia Ruan et al. reported results from a phase II study involving 21 previously untreated MTCL patients receiving 6 cycles of CHOP plus oral azacytidine. More than 80% of the sample had the TFH-phenotype. At the end of therapy, ORR was 76.5% for all patients and 86.7% for cases with TFH-phenotype. Estimates of PFS and OS at 1 year were 61.1% and 88.9%, respectively, for nMTCL-TFH. *TET-2* mutations were associated with higher rates of CR (p=0.014), PFS (p=0.012) and OS (p=0.042). In contrast, *DNMT3A* mutations were associated with decreased OS (p=0.028) (120). On the other hand, in 2015, French researchers published results of a phase Ib/II study testing the combination CHOP plus romidepsin (Ro-CHOP) in 37 previously untreated patients with nMTCL. The regimen was initially shown to be effective and safe, allowing for escalation to a phase III trial (136). However, the results of the phase III study, involving 421 patients with newly diagnosed MTCL (Ro-CHOP = 211, CHOP = 210) have just been released. In addition to being associated with higher rates of grade 3/4 hematologic toxicity, the addition of romidepsin to CHOP did not increase response rates, PFS, and OS. Therefore, the authors concluded that, although based on a biological rationale, the Ro-CHOP association did not represent a significant advance in the standard of care for primary therapy of MTCL (121). **Table 1** summarizes the results of different studies comparing CHOP regimen and other approaches for treatment of AITL in different clinical settings.

**Table 1:** Main studies evaluating alternative therapeutic strategies to the CHOP regimen for MCTL in different clinical settings.

Author	Study design	Population	Therapeutic strategies	Outcomes
Schmitz N, 2010	Retrospective	289 nMTCL (28 AITL)	CHOP vs CHOEP	3y EFS: 50.0 vs 67.5% (for AITL)
Ellin F, 2014	Retrospective	755 nMTCL (104 AITL)	CHOP vs CHOEP	5y PFS: 23.0 vs 40.0% (for AITL)
Lage L, 2022	Retrospective	124 nMTCL (13 AITL)	CHOP vs CHOEP	2y PFS: 69.7 vs 25.0% (for all nMTCL)
Brink M, 2022	Retrospective	1427 nMTCL (294 AITL)	CHOP vs CHOEP	5y OS: 44.0 vs 64.0% (for all nMTCL)
Gallamini A, 2007	Prospective, Phase II	24 MTCL (6 AITL)	CHOP plus alemtuzumab	2y FFS: 48%
Ganjoo K, 2014	Prospective, Phase II	46 MTCL (17 AITL)	CHOP plus bevacizumab	1y PFS: 44% (57% for AITL)
Lemonnier F, 2021	Prospective, Phase II	80 nMTCL (67 AITL)	CHOP plus lenalidomide	2y PFS: 42% for AITL
Meewes FO, 2022	Retrospective	335 AITL	R-CHO(E)P vs R-CHOP	2y PFS: 45.0 vs 40.0%
Horwitz S, 2019	Prospective, Phase III	452 MTCL (> 70% ALCL)	BV-CHP vs CHOP	Median PFS: 48.2 vs 20.8 months
Falchi L, 2021	Prospective, Phase II	25 R/R nMTCL (20 nMTCL-TFH)	5-azacytidine plus romidepsin	Median PFS: 8.0 months
Ruan J, 2020	Prospective, Phase II	21 nMTCL (16 nMTCL-TFH)	5-azacytidine plus CHOP	1y PFS: 56.8% (61.1% for AITL)
Bachy E, 2022	Prospective, Phase III	421 MTCL	CHOP vs Romidepsin plus CHOP (Ro-CHOP)	Median PFS: 10.2 vs 12.0 months

nMTCL, nodal mature T-cell lymphoma; AITL, angioimmunoblastic T-cell lymphoma; EFS, event-free survival; PFS, progression-free survival; OS, overall survival; FFS, failure-free survival; ALCL, anaplastic large-cell lymphoma; nMTCL-TFH, nodal mature T-cell lymphoma with follicular T-helper phenotype; CHOP, cyclophosphamide, doxorubicin, vincristine and prednisone; R, rituximab; BV, brentuximab-vedotin; (E), etoposide; Ro, romidepsin.

The role of consolidation therapy with autologous hematopoietic stem cell transplantation (ASCT) in first remission has been evaluated in different retrospective and prospective studies. The two largest retrospective studies were conducted by the Swedish (*Swedish Lymphoma Registry*) and French (*LYSA study*) groups (116, 137). Both studies excluded patients with ALK1+ ALCL, and evaluated 252 and 269 patients with nodal MTCL, respectively. While the French study did not demonstrate a significant difference in OS and PFS between transplanted and non-transplanted cases, the Swedish study demonstrated that patients undergoing upfront ASCT had superior PFS (HR: 0.56, p=0.002) and increased OS (HR: 0.58, p=0.004) (116, 137). Another large retrospective study was conducted by Abramson JS et al. (2014), aiming to evaluate the optimal frontline therapy for PTCL, as well as assess the impact of upfront ASCT in these malignancies. This trial included 341 newly diagnosed PTCL patients from 2000 to 2011, and 23% of them had a diagnosis of AITL. Most patients (70%) received CHOP-like therapy and only 10% experienced upfront ASCT. Although ORR was 73%, 24% of cases were chemo-refractory and the 3-year PFS estimate was only 32%, significantly lower than DLBCL-matched patients. In this study, early-stage disease and response to initial therapy were the main predictors of favorable outcomes. However, no difference in OS was observed based on choice of primary chemotherapy regimen or consolidation with ASCT in first remission (138).Among prospective studies, the first to assess the role of upfront consolidation with ASCT in PTCL was conducted by Reimer et al. (2008) (139). Eighty-three patients with PTCL were included in this trial, with 32.5% (n=27) having AITL diagnosis, but only 55 (66%) received upfront ASCT consolidation. The main reason for not receiving the transplant was disease progression. ORR was 66% (56% CR and 8% PR). With a median follow-up of 33 months, the estimated 3-year OS, DFS and PFS were 48%, 53%, and 36%, respectively. The results obtained by the German group in this prospective and multicenter trial suggested, for the first time, a substantial impact on outcomes for upfront ASCT in PTCL (139).

The largest prospective study addressing the role of upfront ASCT in PTCL was the *NLG-T-01* trial, conducted by the Nordic group (140). In this phase 2, prospective and multicenter study, 160 patients with non-ALK1+ nodal MTCL were treated with 6 cycles of CHOEP-14. Patients on first CR/PR were consolidated with BEAM (carmustine, etoposide, cytarabine, and melphalan) followed by ASCT. A total of 71% of cases (115/160) underwent ASCT, with estimated 5-year PFS and OS of 44% and 51%, respectively. A significant benefit was particularly seen in the ALK1-negative ALCL group, with 5-year OS and PFS estimates of 70% and 61%, respectively. AITL patients had 5-year OS and PFS estimates of 51% and 49%, respectively (140).

Two other recent large prospective studies evaluated the impact of upfront ASCT in patients with PTCL including AITL cases. In the study conducted by investigators from the COMPLETE group (*Comprehensive Oncology Measures for Peripheral T-Cell Lymphoma Treatment*) 119 patients with non-ALK1-positive nodal PTCL were included (36 experienced ASCT at first CR and 83 did not receive ASCT). With a median follow-up of 2.8 years, the median OS was not reached in patients who underwent ASCT and was 57.6 months in those who did not receive the transplant (p=0.06). ASCT has been associated with increased survival in patients with advanced-stage disease and in those with high-risk IPI, with particular benefit of OS and PFS for AITL cases (141). In the study of the Korean group, 191 patients with PTCL were prospectively enrolled, with 31.4% (n=60) diagnosed with AITL. Among transplant-eligible patients (n=59), 54.2% (n=32) had experienced this therapeutic modality, but there were no significant differences in OS and PFS between the transplanted and non-transplanted arms. However, in patients with AITL, the ASCT promoted PFS benefit, without offering significant differences in OS. Although up-front consolidation with ASCT still offers contradictory results in the therapeutic management of PTCL in the modern era, data from both studies suggest that ASCT may provide a real survival benefit in AITL, particularly when applied in selected cases (142).

Few studies have evaluated the role of upfront allogeneic stem cell transplantation (alloSCT) in nodal MTCL. In this sense, the *AATT* study was the only phase 3 trial to assess the role of upfront stem cell transplantation in patients with nodal MTCL, excluding cases of ALK1+ ALCL. This study compared outcomes of patients undergoing first-line ASCT and alloSCT. The study was prematurely discontinued because the interim analysis revealed a high-rate of transplant-related mortality (TRM) in the alloSCT arm. After a follow-up of 42 months, the 3-year EFS was 43% and 38% for alloSCT and ASCT, respectively. The 3-year OS was 57% and 70% for alloSCT and ASCT, respectively. Although no patient who underwent alloSCT relapsed versus 36% (13/36) in the ASCT group, the TRM was 31% (8/26) in the alloSCT group versus 0% in the ASCT group (0/41) (143).

In the context of R/R disease, the therapeutic strategy for AITL patients should be determined in view of the objective of the treatment, whether with curative or palliative intent. Patients with R/R disease treated with curative intuit are usually rescued with polychemotherapeutic regimens based on platinum derivatives, such as DHAP (dexamethasone, cytarabine, cisplatin), DHAOX (dexamethasone, cytarabine, oxaliplatin), ICE (ifosfamide, carboplatin, etoposide), ESHAP (etoposide, methylprednisolone, cytarabine and cisplatin), GDP (gemcitabine, dexamethasone and cisplatin) or GEMOX (gemcitabine, oxaliplatin) as bridges to ASCT (if not previously submitted to this therapeutic strategy) or alloSCT (if previously experienced ASCT). In this context, in general, the 5-year OS estimate with ASCT is around 45-53%, with TRM oscillating between 0-10% (144–147). For the alloSCT, the 5-year OS ranges from 50-81%, with 5-year PFS ranging from 40-64%, and TRM between 12-33%, according to different studies (146, 148–150). Patients with R/R disease who will be treated with non-curative intent should preferably experience regimens with agents that do not cause cumulative toxicity, in low-doses and “non-finite”. For this purpose, several options may be considered, including monotherapy with romidepsin, belinostat, 5-azacytidine, pralatrexate, bendamustine, brentuximab-vedotin, lenalidomide and cyclosporine A, with ORR ranging from 8-75%, and a short median duration of response (3.5-17 months) (110). However, we must highlight that some of these compounds are not approved by many regulatory agencies for AITL management; therefore, the availability of these products might vary in different countries. Among combination regimens tested in phase 1/2 studies, the most promising appear to be romidepsin/pralatrexate (ORR 71%; CR 29%), oral 5-azacitidinb/romidepsin (ORR 71%; CR 71%) and gemcitabine/copanlisib (ORR 72%; CR 32%) (135, 151, 152).

**Figure 5:**
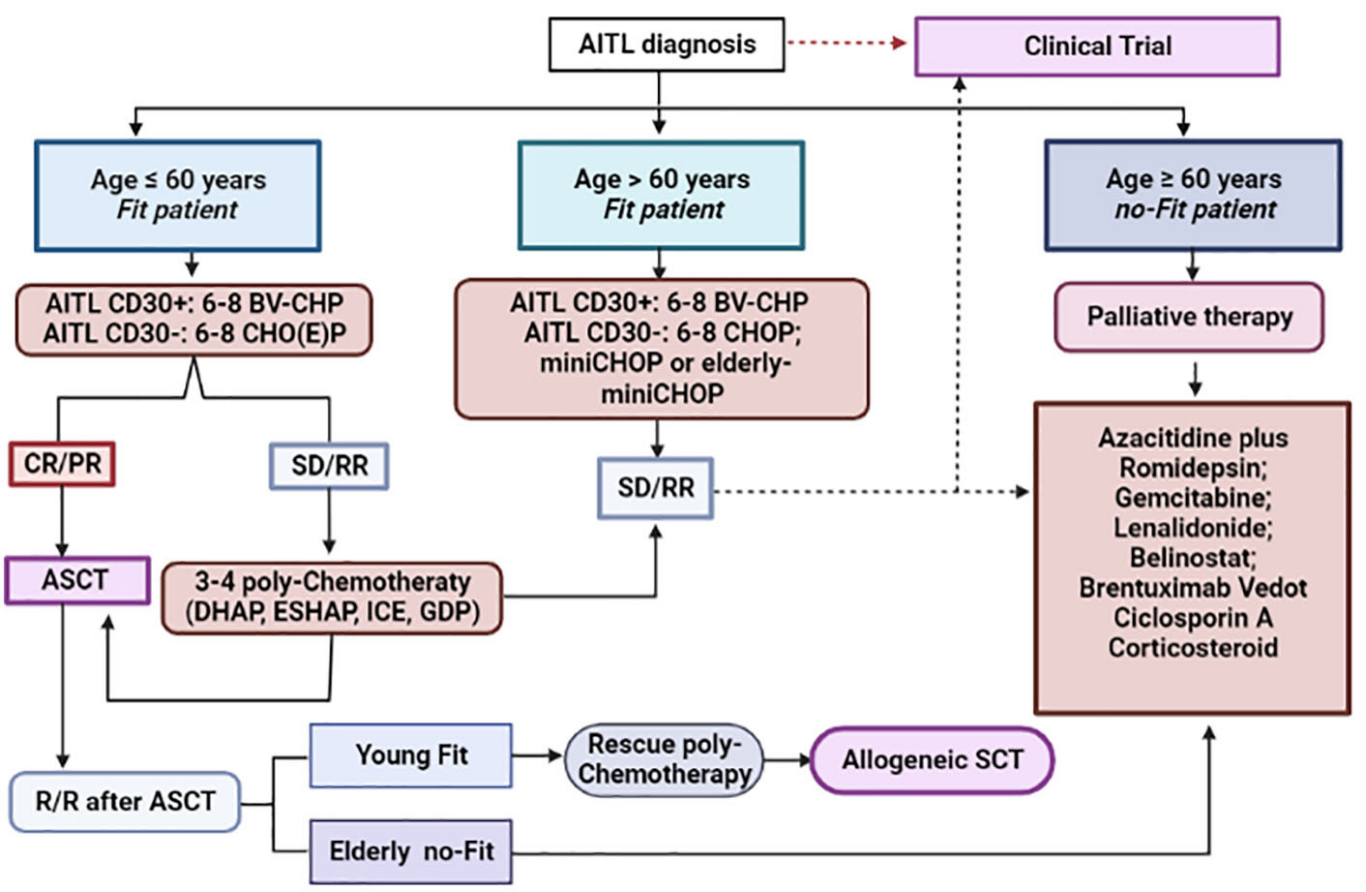
Therapeutic algorithm for the management of AITL patients. Up-front therapy and treatment of relapsed/refractory cases. *AITL, angioimmunoblastic T-cell lymphoma; BV-CHP, brentuximab-vedotin plus cyclophosphamide, doxorubicin and prednisone; CHO(E)P, cyclophosphamide, doxorubicin, vincristine, etoposide and prednisone; CR, complete response; PR, partial response; SD, stable disease; R/R, relapsed/refractory disease; ASCT, autologous stem cell transplantation; DHAP, dexamethasone, high-doses of cytarabine, cisplatin; ESHAP, etoposide, methylprednisolone, high-doses of cytarabine, cisplatin; ICE, ifosfamide, carboplatin, etoposide; GDP, gemcitabine, dexamethasone and cisplatin.

Although chimeric antigen T-cell receptor (CAR-T) therapy has proved to be an interesting form of immunotherapy in several B-cell lymphoid malignancies, such as R/R diffuse large B-cell lymphoma, follicular lymphoma, B-cell acute lymphoblastic leukemia and multiple myeloma, its use has been extremely limited in the setting of PTCL. While encouraging results were seen with some novel agents, there is still very few data about CAR-T therapy in PTCL; limited experience and lack of controls preclude critical analyses. One of the main challenges in the use of CAR-T therapy in T-cell malignancies is due to the fact that neoplastic cells share a series of common antigens with normal T-lymphocytes, which can lead to fratricide and serious T-cell lymphoid aplasia in the receptor. To mitigate this effect, a selection of appropriate antigenic targets is essential. Currently, specific antigens have been selected for the construction of chimeric products, among which CD30, CD37, TRBC1, CCR4 and CCR9 stand out. Use of nanobody-derived or naturally selected CAR-T are attractive strategies to overcome fratricide. Another problem intrinsic to the use of this therapeutic modality in T-cell malignancies refers to the potential contamination of the product collected for the construction of CAR-T with clonal T-cells, however, the use of allogeneic CAR-T products or CAR-NK-cells are possible strategies with ability to mitigate this contamination. Currently, data about the use of CAR-T therapy in PTCL, particularly for AITL are very scarce, although it may constitute an interesting therapeutic option for R/R disease in the next future (153). **Figure 5** summarizes the proposal for a therapeutic algorithm to approach patients with AITL in first line and in the context of R/R disease.

## 8 Conclusion

AITL is a peculiar subtype of nodal MTCL derived from monoclonal proliferation of TFH-cells and is associated with a poor overall prognosis. Recent studies, involving modern technologies, such as GEP and NGS, have contributed to determine its cell of origin, define its unique gene-signature, and revealed a specific-pattern of recurrent mutations, which have contributed in a decisive way for its biological understanding. Studies conducted in xenograft models have elucidated the multi-step neoplastic transformation process of AITL, from acquisition of premalignant epigenetic mutations to the development of disease-specific mutations (“driver-mutations”), such as *RhoA* G17V. However, such biological progress has not yet been translated objectively into the therapeutic field. Currently, the treatment of patients with AITL is still based on the wide use of anthracyclic agents, with dismal outcomes, being an unmet medical need. Hopefully, new agents, with employment based on biological rationale, such as HMAs and HDAi may represent a paradigm shift in the approach to this lymphoma in the near future. For this, collaborative multicenter studies involving large cohorts from different parts of the globe are necessary.

## Author contributions

LL, HC, CR, SdS, and JP reviewed the literature, organized, and wrote the article. All authors contributed to the article and approved the submitted version.
